# Analysis of Kinetoplast DNA from Mexican Isolates of *Leishmania (L.) mexicana*

**DOI:** 10.1155/2012/279081

**Published:** 2012-12-25

**Authors:** Omar Hernández-Montes, Saúl González Guzmán, Federico Martínez Gómez, Douglas C. Barker, Amalia Monroy-Ostria

**Affiliations:** ^1^Department of Immunology, National School of Biological Sciences, National Polytechnic Institute, 11340 México City, Mexico; ^2^Department of Parasitology, National School of Biological Sciences, National Polytechnic Institute, 11340 México City, Mexico; ^3^Christ's College, Cambridge CB2 3BU, UK

## Abstract

This study analyzed DNA minicircles of Mexican isolates of *L. (Leishmania) mexicana* to look for genetic differences between strains isolated from patients with diffuse cutaneous (DCL) and localized (LCL) leishmaniasis. The kDNA was analyzed using polymerase chain reaction (PCR), restriction fragment polymorphism analysis of the PCR products (PCR-RFLP) and the PCR products were sequenced. In the PCR with primers specific for the subgenus *Leishmania*, the Mexican isolates gave higher amplification products than the other *L. mexicana* complex strains and with specific primers for the *L. mexicana* complex they were poorly amplified. In the PCR-RFLP analysis with the *Eco RV*, *Hae III*, and *Mbo I* endonucleases, the Mexican isolates displayed similar restriction patterns, but different from the patterns of the other members of the *L. mexicana* complex. In the phylogenetic tree constructed, the kDNA sequences of the Mexican clones formed two groups including sequences of LCD or LCL clones, apart from the other *L. mexicana* complex members. These results suggest that the kDNA minicircles of the Mexican isolates are more polymorphic than the kDNA of other members of the *L. mexicana* complex and have different recognition sites for the restriction enzymes used in this study.

## 1. Introduction

Cutaneous leishmaniasis (CL) is the most widespread form of leishmaniasis, causing primary localized skin lesions (LCL) from which parasites can disseminate to the nasopharyngeal mucosa and cause the disfiguring lesions typical of mucocutaneous leishmaniasis (MCL) or disseminated to the entire body as nodular lesions (DCL) [1].

American cutaneous leishmaniasis is characterized by a spectrum of clinical presentations. These include LCL caused by *L. (Leishmania) mexicana*, DCL caused by *L. (Leishmania) amazonensis*, *L. (Leishmania) venezuelensis,* and *L. (Leishmania) pifanoi*, all belonging to the *Leishmania mexicana* complex, and MCL caused by members of the *Leishmania braziliensis* complex [2].

Cutaneous leishmaniasis in Mexico is highly endemic with broad geographical distribution of the different clinical manifestations. In endemic areas, LCL or MCL can be caused by members of both *L. mexicana* and *L. braziliensis* complexes [3], making more accurate analysis and identification of the *Leishmania* strains imperative so that opportune and appropriate treatment can be administered.

Polymerase chain reaction (PCR) approaches have been used in parasite DNA analysis. Several PCR molecular targets have been developed, including minicircle kinetoplast DNA (kDNA) [3], the miniexon (spliced leader RNA) gene [4], and the gp63 PCR-RFLP [5], among others.

Kinetoplast DNA contains approximately 10,000 minicircles per cell that are around 800 base pairs (bp) in size, with a roughly 200 bp conserved region and an approximately 600 bp variable region [6, 7]. The sequence classes of minicircles are organized in a uniform scheme and contain one or several highly conserved regions (CR). There are three highly conserved blocks (CSB) within CRs: CSB1 (GGGCGT), CSB2 (CCCCGTTC), and CSB3 (GGGGTTGGTGTA) known as the universal minicircle sequence (UMS) [6, 7]. Minicircles are subjected to frequent insertions and deletions leading to diversity in size; removal and insertion of recognition sites for various restriction enzymes and some size heterogeneity in the overall minicircle size can be observed [8].

In the present study, the analysis of kDNA minicircles of isolates of *L. (L.) mexicana* from skin lesions of patients with DCL or LCL was performed in order to determine whether a particular minicircle class is exclusive of one strain of *Leishmania *or the differences in clinical manifestation of the disease are related to any particular minicircles classes.

## 2. Materials and Methods 

### 2.1. *Leishmania* Species and Culture Conditions

This study was reviewed and approved by the Ethics Committee of the Escuela Nacional de Ciencias Biologicas, Instituto Politecnico Nacional, Mexico, in agreement with international ethics guidelines for biomedical research involving human subjects (Ley General de Salud, Mexico).

**Figure 1:**
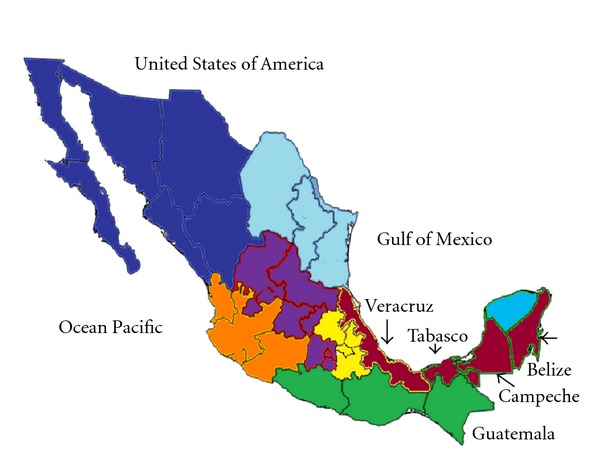
Map of Mexico, showing the endemic regions studied in this work, Veracruz, Tabasco, and Campeche states.

The study was conducted with seven Mexican isolates of *L. (L.) mexicana* from patients with multiple nonulcerative nodular skin lesions (DCL) or with ulcerative skin lesions developing at the site of the sandfly bite (LCL) from different states in Mexico (Figure 1), included after informed consent was obtained. For parasite isolation, needle aspirates were taken from the edge of cutaneous lesions prior to patients receiving treatment. The Mexican isolates were sent to the London School for Tropical Medicine and Hygiene (UK) for isoenzyme characterization. Reference strains of members of the *L. mexicana* complex were also included (Table 1). 

The strains of *Leishmania* were cultured in RPMI medium supplemented with 10% fetal calf serum at 28°C.

### 2.2. DNA Extraction

DNA was prepared, as described elsewhere [3], by centrifuging 10^9^ parasites of an exponential phase of growth culture at 1900 g for 10 min. The pellet was resuspended in 1 mL of NET 100 (100 mMTris-HCl, pH 8.0; 100 mM EDTA; 100 mM NaCl), 1% SDS, and 4 *μ*L of 10 mg/mL proteinase K (Sigma) and incubated at 37°C overnight, followed by two phenol-chloroform extractions and ethanol precipitation. The DNA extracted was washed with 70% ethanol and dissolved in 100 *μ*L distilled water and kept at −70°C until use.

### 2.3. Polymerase Chain Reaction

The universal primers AJS1 (GGGGTTGGTGTAAAATAG) and DeB8 (CCAGTTTCCCGCCCCG), specific for kDNA minicircles of the *Leishmania* subgenus [9], and the M1 (CCAGTTTCGAGCCCCGGAG) and M2 (GGTGTAAAATAGGGCCGGATGCTC) primers, specific for minicircles of the *L. mexicana* complex [10] were used to amplify kDNA from reference strains and from Mexican isolates. PCR amplifications were done as described elsewhere [9, 10], in a solution of 0.2 mM each of deoxyribonucleotide (Invitrogen Life Technologies, Carlsbad, CA, USA), 50 pmol of each specific primer, 2.5 units of Taq DNA polymerase (Perkin Elmer Cetus), 100 ng of DNA template, 1.5 mM of MgCl_2_ in a final volume of 100 *μ*L. Samples were denatured at 96°C for 6 min. PCR (35 cycles) consisted of annealing at 60°C for AJS1 and DeB8 primers and 67.5°C for M1 and M2 primers, extension at 72°C for 1 min, and final extension at 72°C for 10 min on a Perkin-Elmer Model 2400 thermal cycler (Perkin Elmer, Roche Molecular Systems Inc., Brancbourg, NJ, USA). PCR products (10 *μ*L) were fractionated by electrophoresis in 1% agarose or 8% acrylamide gels in TBE (90 mMTris-HCl pH 8.3, 90 mM boric acid, and 2 mM EDTA) stained with ethidium bromide (50 g/mL) and were observed under a transilluminator (SIGMA Chemical Co., St. Louis, MO, USA).

### 2.4. Restriction Fragment Polymorphism Analysis of kDNA

The kDNA amplicons obtained with the AJS1 and DeB8 primers of the *Leishmania* reference strains and the Mexican isolates were digested with the *Bam HI*, *Eco RV*, *Hae III*, *Hind III*, *Kpn I*, *Mbo I*, *Mse I*, *Msp I*, and *Xba I* endonucleases (Gibco BRL). The restrictions were performed following the supplier's instructions, briefly: 10 *μ*g of PCR product, 2.5 *μ*L of the correspondent buffer, and 10 U of the endonuclease in a final volume of 25 *μ*L were incubated at 37°C for 2 h; the *Taq I* was incubated at 67°C. The reactions were analyzed by electrophoresis as mentioned above.

### 2.5. Sequencing of Minicircles of Mexican Isolates

The kDNA amplicons of the Mexican isolates, MC (from patient with LCL), and GS (from patient with DCL) obtained with the AJS1 and DeB8 primers were gel purified using a QIA quick gel extraction Kit (Qiagen, Germany), ligated into a TA Cloning vector (pCR II Vector) (Invitrogen Life Technologies), and transformed into *Escherichia coli* INV *α*F′. Plasmid DNA was extracted using the Wizard Plus Minipreps DNA Purification System (Promega Corporation, Madison, WI, USA) and sequenced. Nucleotide sequences were determined using a dideoxynucleotide chain termination sequence kit (ABI PRISM Dye Terminator Cyclers Sequencing Ready Reaction Kit, Perkin Elmer) and an Abi Prism M 310 Genetic Analyzer automated sequencer (Perkin Elmer).

### 2.6. Sequence Alignments And Phylogenetic Analysis

The sequences were edited with the DNAMAN, Chromas version 2.0, and Seaview software [11]. Multiple sequences were aligned using Clustal-X Ver. 1.83 [12]. An unrooted phylogenetic tree was constructed using the neighbor-joining method [13] with the Clustal-X program. Evolutionary distances were calculated using Kimura's two-parameter method [14], with MEGA (Molecular Evolutionary Genetics Analysis), Version 3.1. [15]. A total of 1000 bootstrapping replicates were used for the neighbor-joining analysis to obtain relative support for internal nodes. The kDNA sequence data were compared with the sequences of the other members of the *L. mexicana* complex previously published in GenBank.

## 3. Results

**Figure 2:**
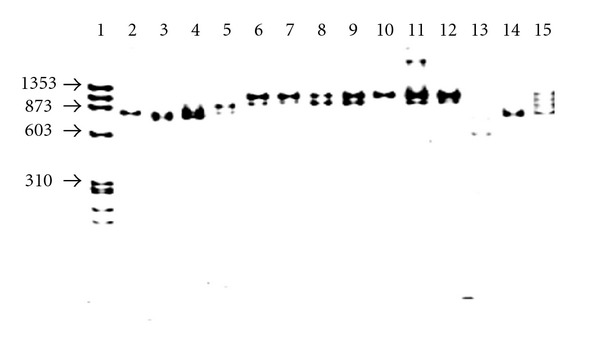
PCR Amplification of kDNA with the primers AJS 1 and DeB 8. Lane 1, MWMX174 Hae III; 2, *L. (L.) granhami* JAP78; 3, *L. (L.) granhami* HM76; 4, *L. (L.) mexicana* M379; 5, *L. (L) mexicana* BEL 21; 6, *L. (L) mexicana* SOB; 7, *L. (L.) mexicana* YUC; 8, *L. (L.) mexicana* MC; 9, *L. (L.) mexicana* GS; 10, *L. (L.) mexicana* HF; 11, *L. (L.) mexicana* AM; 12, *L. (L.) mexicana* AG; 13, *L.(L.) amazonensis* M2269; 14, *L. (L.) amazonensis* PH8; 15, *L. (L.) pifanoi* L11.

PCR specific for the *Leishmania* subgenus performed with the AJS1 and DeB8 primers resulted in the amplification of the kDNA of *L. (L.) mexicana* BEL21 and *L. (L.) mexicana* M379, the reference strains, and the Mexican isolates (lanes 6–12) giving 700 to 870 bp amplification bands; *L. (L.) amazonensis* M2269 and PH8 as well as *L. (L.) garnhami* HM76 and JAP78 gave bands less than 700 bp (Figure 2).

PCR specific for the *L. mexicana* complex, with the primers M1 and M2, resulted in the amplification of the *L. (L.) amazonensis* reference strain, giving 700 bp amplification bands; the other references strains gave 800 bp bands. The Mexican isolates were poorly amplified, giving 680 bp bands (data not shown). 

**Figure 3:**
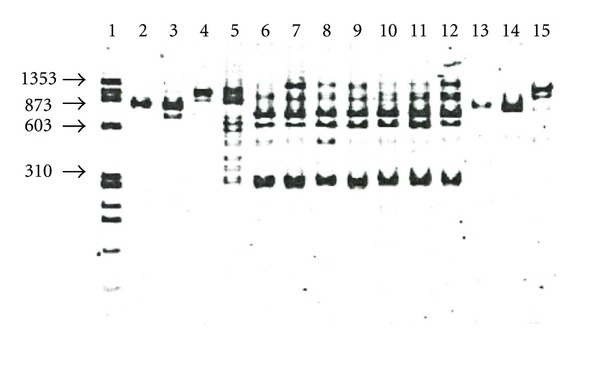
PCR-RFLP analysis with the endonuclease *Eco RV*. Lane 1, MW X174 *Hae* III; 2, *L. (L.) garnhami* JAP78; 3, *L. (L.) garnhami* HM76; 4, *L. (L.) mexicana* M379; 5, *L. (L.) mexicana* BEL 21; 6, *L. (L.) mexicana* SOB; 7, *L. (L.) mexicana* YUC; 8, *L. (L.) mexicana* MC; 9, *L. (L.) mexicana* GS; 10, *L. (L.) mexicana* HF; 11, *L. (L.) mexicana* AM; 12, *L. (L.) mexicana* AG; 13, *L. (L.) amazonensis* M2269; 14, *L. (L.) amazonensis* PH8; 15, *L. (L.) pifanoi* LL1.

**Figure 4:**
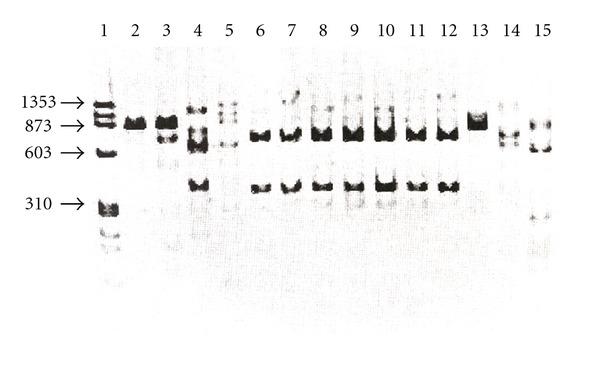
PCR-RFLP analysis with the endonuclease *Mbo I*. Lane 1, MW X174 *Hae* III; 2, *L. (L.) garnhami* JAP78; 3, *L. (L.) garnhami* HM76; 4, *L. (L.) mexicana* M379; 5, *L. (L.) mexicana* BEL 21; 6, *L. (L.) mexicana* SOB; 7, *L. (L.) mexicana* YUC; 8, *L. (L.) mexicana* MC; 9, *L. (L.) mexicana* GS; 10, *L. (L.) mexicana* HF; 11; *L. (L.) mexicana* AM; 12, *L. (L.) mexicana* AG; 13, *L. (L.) amazonensis* M2269; 14, *L. (L.) amazonensis* PH8; 15, *L. (L.) pifanoi *LL1.

Restriction length polymorphism analysis. In endonuclease digestion of kDNA amplicons with the *Eco RV* endonuclease, the Mexican isolates displayed a six band pattern (lanes 6–12), the reference strain *L. (L.) mexicana *Bel21 showed eight bands (lane 5), the other *Leishmania* strains showed two bands and some were not restricted (Figure 3). With *Hae III* the Mexican isolates all displayed the same pattern, which was several bands in length, the *L. mexicana* complex reference strains also displayed a restriction pattern of several bands, but a different size (data not shown). With *Mbo I*, all the Mexican isolates displayed a similar two-band restriction pattern (lanes 6–12) and the *Leishmania* reference strains showed a pattern of two or four bands (Figure 4). With the *Xba I* only, the Mexican isolates were restricted to displaying patterns several bands long (data not shown); the *Hind III*, *MspI*, and *Spe I* endonucleases restricted only certain strains, but not the Mexican isolates (data not shown). The *Bam HI* and *Kpn I* endonucleases did not digest any *Leishmania* strains (data not shown). 

**Figure 5:**
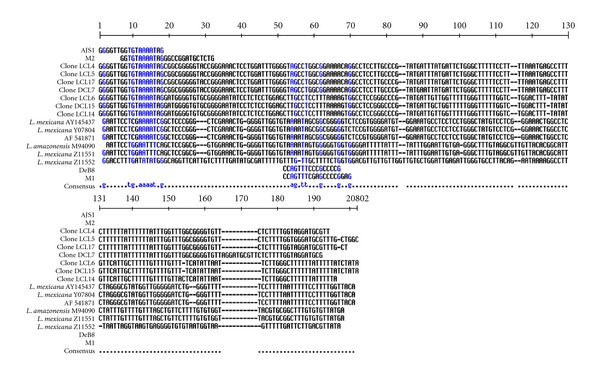
Aligment of the kDNA minicircles sequences classes (from 1 to 200 bp). Clones of the Mexican isolates: LCL4, DCL7, LCL17, DCL15, LCL6, and LCL14. Sequences of other members of the *Leishmania mexicana *complex published in the GeneBank: *L. mexicana*Y07807, AF54187, Y145437; *L. (L.) amazonensis* M 94090; *L. (L.) mexicana* Z11551, Z11552, Z11554, Z11556, and Z11549; *L. (L.) amazonensis* M21325, M 21327; *L. (L.) mexicana* Z11553, Z11555.

In the cloning and sequencing of kDNA minicircles of the Mexican isolates, seven minicircle clone sequences were obtained, five from the LCL isolated and two from the DCL (data not shown).All seven clones showed high homology in their conserved part, presenting the highly conserved block CSB3 (GGGGTTGGTGTA) known as the universal minicircle sequence (UMS) (Shlomai, 2004), but after position 19 two groups of minicircle sequences have been found, one 757–759 bp (LCL6, LCL14, and LCD15), which presents the ACTCCTGGATTT motif, and the 790 to 791 bp group (LCD7, LCL17, LCL5, and LCL4), which presents the TATCCTCTCCT motif. The comparison of the sequences of these two groups revealed differences in deletions and substitutions of bp along the kDNA minicircle (Figure 5).

**Figure 6:**
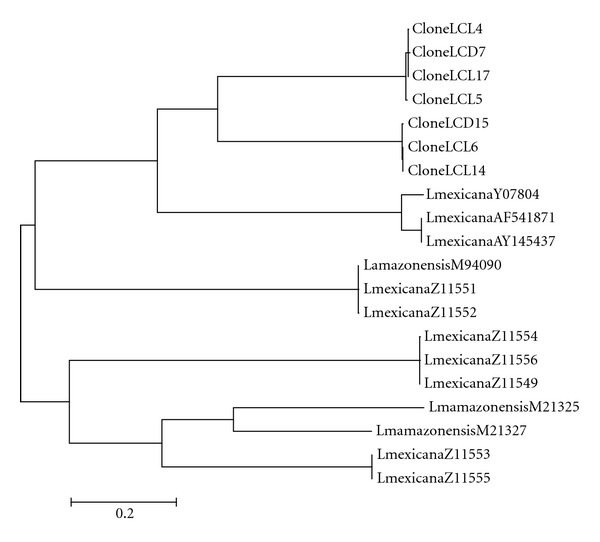
Phylogenetic tree constructed with sequences of kDNA minicircles. *Leishmania mexicana *complex members published in theGenBank:* L. (L.) mexicana *Y07807, AF54187, Y145437; *L. (L.) amazonensis* M 94090; *L. (L.) mexicana* Z11551, Z11552, Z11554, Z11556, and Z11549; *L. (L.) amazonensis* M21325, M 21327; *L. (L.) mexicana* Z11553, Z11555; clones of *L. (L.) mexicana. *Mexican isolates clones: LCL4, DCL7,LCL 17, LCL5, DCL15, LCL6, and LCL14.

Based on sequence alignments of kDNA minicircles of Mexican isolates and the other members of the *L. mexicana* complex, phylogenetic trees were constructed. The consensus tree showed nine groupings. The first and second groups were formed with LCL and DCL Mexican clones. A further two groups were formed with clones of *L. (L.) mexicana *from international strains. Another two groups were formed with *L. (L.) amazonensis* and *L. (L.) mexicana* clones. Another node was formed with two groups including only *L. (L.) amazonensis* clones. The last group was formed with clones of *L. (L.) mexicana* (Figure 6).

## 4. Discussion

In the amplification of kDNA with the primers specific for the *Leishmania* subgenus, the Mexican isolates showed PCR products that had a slightly higher molecular weight than the *L. (L.) Mexicana *M379,* L. (L.) mexicana Bel21*, *L. (L.) amazonensis*, and *L. (L.) pifanoi* reference strain PCR products (Figure 2). On the other hand, with the specific primers for the *L. mexicana *complex none of the Mexican isolates were properly amplified as the other members of the *L. mexicana* complex had been (data not shown). These results could be due to the fact that the specific primers for *Leishmania* subgenus have the UMS sequence as do the Mexican isolates, and the sequences of the M1 and M2 primers have less homology with the Mexican isolates than with the other members of the *L. mexicana* complex, indicating that the kDNA minicircles of the Mexican isolates are different from the other members of the *L. mexicana* complex.

Interestingly, the Mexican isolates digested with the *Eco RV *(Figure 3), *Hae III* (data not shown), and *Mbo I* endonucleases (Figure 4) displayed similar restriction patterns between them, totally different from the digestion patterns displayed for the other members of the *L. mexicana* complex. Furthermore, with the *Xba I* endonuclease only the Mexican isolates were restricted (data not shown). These results clearly suggest that the kDNA minicircles of the Mexican isolates are more polymorphic than the kDNA of the other members of the *L. mexicana* complex and have different recognition sites for the several restriction enzymes used in this study. Furthermore, in studies of PRC-RFLP of kDNA [16] found six basic digestion patterns in Mexican isolates of *L. (L.) mexicana* that were different from the digestion patterns displayed for the other members of the *L. mexicana *complex reference strains. 

The Mexican isolate clone sequence classes of minicircles formed two groups including DCL and LCL clones in each group, indicating that a particular minicircle class is not exclusive of one strain of *Leishmania,* furthermore the different clinical manifestation of the disease (LCL or DCL) is not related to any particular minicircle classes (Figure 5). Comparing these sequences with the sequences of other members of the *L. mexicana* complex, it was observed that they did not have extensive sequence homology. These results agree with Barker's studies [17] showing that the minicircles of representative strains of the main complexes of *Leishmania* do not share extensive sequence homology. 

 In the phylogenetic tree constructed with these sequences, the other members of the *L. mexicana complex* formed groups with *L. (L.) mexicana* and *L. (L.) amazonensis* clones apart from the groups formed exclusively with the Mexican clones. These may indicate that the Mexican isolates have sequence classes of minicircles that are different from the other members of the *L. mexicana* complex isolated in other countries (Figure 6).

On the other hand, Gutiérrez-Solar et al. [18] found greater homology between minicircles of geographically separated isolates than minicircles of the same stock. Within human species of *Leishmania*, Rogers and Wirth [19] described highly homogeneous sequences only in minicircles of geographically related isolates. 

On the other hand, in studies with *L. (L.) amazonensis* resistant to tunicamycin, Lee et al. [20] found that certain minor minicircle sequence were selected to replicate and to replace the dominant type of minicircles and become predominant.

Although *Leishmania *kDNA is very polymorphic, the PCR of kDNA analysis is a very useful and sensitive enough for detecting *L. donovani* genes in skin biopsy specimens from patients and is recommended as a confirmatory diagnostic tool for PKDL [21], and for characterization of cutaneous isolates of *Leishmania* by isoenzyme typing combined with kDNA restriction analysis [22].

 In conclusion it seems that the minicircle classes could change in the kDNA network [8]. We cannot say that one or another minicircle sequence class within the kDNA network is permanent or typical of a virulent or less virulent *Leishmania* strain, and the clinical manifestation of the disease (LCL or DCL) is not related to any particular minicircle classes. Furthermore there is no relation with their geographical distribution.

## Figures and Tables

**Table 1a:** (a)

Reference strains	International code^1^	Source
*L*. (*L*.) *mexicana *	MHOM/BZ/62/BEL21	1
*L*. (*L*.) *mexicana *	MHOM/BZ/62/M379	1
*L*. (*L*.) *amazonensis *	IFLA/BR/67/PH8	1
*L*. (*L*.) *amazonensis *	MHOM/BR/73/M2269	1
*L*. (*L*.) *garnhami *	MHOM/VE/75/HM76	1
*L*. (*L*.) *garnhami *	MHOM/VE/76/JAP78	1
*L*. (*L*.) *pifanoi *	MHOM/VE/57/LL1	1

^1^: The WHO *Leishmania*  Reference Collection at the London School of Hygiene and Tropical Medicine, London, UK.

**Table 1b:** (b)

Mexican isolates^2^		Origin
From patients with LCL		
*L*. (*L*.) *mexicana *	MHOM/MX/88/HRC JS (SOB)	Tabasco
*L*. (*L*.) *mexicana *	MHOM/MX/88/HRC MC (MC)	Tabasco
*L*. (*L*.) *mexicana *	MHOM/MX/83/UAYV (YUC)	Campeche

From patients with DCL		
*L*. (*L*.) *mexicana *	MHOM/MX/84/ISET GS (GS)	Tabasco
*L*. (*L*.) *mexicana *	MHOM/MX/85/ISET HF (HF)	Veracruz
*L*. (*L*.) *Mexicana *	MHOM/MX/92/INDRE AM (AM)	Veracruz
*L*. (*L*.) *mexicana *	MHOM/MX/92/INDRE AG (AG)	Tabasco

^2^: Instituto Nacional de Diagnostico y Referencia Epidemiologicos, Secretaria de Salud, Mexico. Tabasco, Veracruz, and Campeche states are located in the Mexican Southeast.

## References

[1] Reithinger R, Dujardin JC, Louzir H, Pirmez C, Alexander B, Brooker S (2007). Cutaneous leishmaniasis. *The Lancet Infectious Diseases*.

[2] Lainson R (1983). The American leishmaniasis: some observations on their ecology and epidemiology. *Transactions of the Royal Society of Tropical Medicine and Hygiene*.

[3] Hernández-Montes O, Monroy-Ostria A, McCann S, Barker DC (1998). Identification of Mexican *Leishmania* species by analysis of PCR amplified DNA. *Acta Tropica*.

[4] Fernandes O, Murthy VK, Kurath U, Degrave WM, Campbell DA (1994). Mini-exon gene variation in human pathogenic *Leishmania* species. *Molecular and Biochemical Parasitology*.

[5] Victoir K, De Doncker S, Cabrera L (2003). Direct identification of *Leishmania* species in biopsies from patients with American tegumentary leishmaniasis. *Transactions of the Royal Society of Tropical Medicine and Hygiene*.

[6] Ray DS (1989). Conserved sequence blocks in kinetoplast minicircles from diverse species of trypanosomes. *Molecular and Cellular Biology*.

[7] Shlomai J (2004). The structure and replication of kinetoplast DNA. *Current Molecular Medicine*.

[8] Singh N, Curran MD, Middleton D, Rastogi AK (1999). Characterization of kinetoplast DNA minicircles of an Indian isolate of *Leishmania donovani*. *Acta Tropica*.

[9] Smyth AJ, Ghosh A, Hassan MQ (1992). Rapid and sensitive detection of *Leishmania* kinetoplast DNA from spleen and blood samples of kala-azar patients. *Parasitology*.

[10] Eresh S, McCallum SM, Barker DC (1994). Identification and diagnosis of *Leishmania mexicana* complex isolates by polymerase chain reaction. *Parasitology*.

[11] Galtier N, Gouy M, Gautier C (1996). SEAVIEW and PHYLO_WIN: two graphic tools for sequence alignment and molecular phylogeny. *Computer Applications in the Biosciences*.

[12] Thompson JD, Gibson TJ, Plewniak F, Jeanmougin F, Higgins DG (1997). The CLUSTAL X windows interface: flexible strategies for multiple sequence alignment aided by quality analysis tools. *Nucleic Acids Research*.

[13] Saitou N, Nei M (1987). The neighbor-joining method: a new method for reconstructing phylogenetic trees.. *Molecular Biology and Evolution*.

[14] Kimura M (1980). A simple method for estimating evolutionary rates of base substitutions through comparative studies of nucleotide sequences. *Journal of Molecular Evolution*.

[15] Baxavanis AD, Ouellette BF (2001). Phylogenetic analysis. *Bioinformatics. A Practical Guide to the Analysis of Genes and Proteins*.

[16] Berzunza-Cruz M, Bricaire G, Romero SZ (2000). *Leishmania mexicana mexicana*: genetic heterogeneity of Mexican isolates revealed by restriction length polymorphism analysis of kinetoplast DNA. *Experimental Parasitology*.

[17] Barker DC (1987). DNA diagnosis of human leishmaniasis. *Parasitology Today*.

[18] Gutiérrez-Solar B, Smyth AJ, Alvar J, Barker DC (1995). *Leishmania infantum*: sequence homology within minicircle classes regardless of geographical distance. *Experimental Parasitology*.

[19] Rogers WO, Wirth DF (1988). Generation of sequence diversity in the kinetoplast DNA minicircles of *Leishmania mexicana* amazonensis. *Molecular and Biochemical Parasitology*.

[20] Lee ST, Liu HY, Lee SP, Tarn C (1994). Selection for arsenite resistance causes reversible changes in minicircle composition and kinetoplast organization in *Leishmania mexicana*. *Molecular and Cellular Biology*.

[21] Nasreen SA, Hossain A, Paul SK (2012). PCR-based detection of *Leishmania* DNA in skin samples of post kala-azar dermal leishmaniasis patients from an endemic area of Bangladesh. *Japanese Journal of Infectious Diseases*.

[22] Urbano J, Sanchez-Moreno ME, Ovalle CE (2011). Characterization of cutaneous isolates of *Leishmania* in Colombia by isoenzyme typing and kDNA restriction analysis. *Revista Ibero-Latinoamericana de Parasitología*.

